# Meta-analysis of tRNA derived RNA fragments reveals that they are evolutionarily conserved and associate with AGO proteins to recognize specific RNA targets

**DOI:** 10.1186/s12915-014-0078-0

**Published:** 2014-10-01

**Authors:** Pankaj Kumar, Jordan Anaya, Suresh B Mudunuri, Anindya Dutta

**Affiliations:** Department of Biochemistry and Molecular Genetics, University of Virginia School of Medicine, Charlottesville, VA 22901 USA; Department of Computer Science and Engineering, Grandhi Varalakshmi Venkatarao Institute of Technology (GVIT), Bhimavaram, Andhra Pradesh 534207 India; ᅟ, PO Box 800733, 1340 Jefferson Park, Ave Jordan Hall Room 1232, Charlottesville, VA USA

**Keywords:** Small RNA, Non-coding RNA, Regulatory RNA, tRF, tRNA

## Abstract

**Background:**

tRFs, 14 to 32 nt long single-stranded RNA derived from mature or precursor tRNAs, are a recently discovered class of small RNA that have been found to be present in diverse organisms at read counts comparable to miRNAs. Currently, there is a debate about their biogenesis and function.

**Results:**

This is the first meta-analysis of tRFs. Analysis of more than 50 short RNA libraries has revealed that tRFs are precisely generated fragments present in all domains of life (bacteria to humans), and are not produced by the miRNA biogenesis pathway. Human PAR-CLIP data shows a striking preference for tRF-5s and tRF-3s to associate with AGO1, 3 and 4 rather than AGO2, and analysis of positional T to C mutational frequency indicates these tRFs associate with Argonautes in a manner similar to miRNAs. The reverse complements of canonical seed positions in these sequences match cross-link centered regions, suggesting these tRF-5s and tRF-3s interact with RNAs in the cell. Consistent with these results, human AGO1 CLASH data contains thousands of tRF-5 and tRF-3 reads chimeric with mRNAs.

**Conclusions:**

tRFs are an abundant class of small RNA present in all domains of life whose biogenesis is distinct from miRNAs. In human HEK293 cells tRFs associate with Argonautes 1, 3 and 4 and not Argonaute 2 which is the main effector protein of miRNA function, but otherwise have very similar properties to miRNAs, indicating tRFs may play a major role in RNA silencing.

**Electronic supplementary material:**

The online version of this article (doi:10.1186/s12915-014-0078-0) contains supplementary material, which is available to authorized users.

## Background

Small RNAs have been defined as 19 to 31 nucleotide long RNAs present primarily in metazoans and plants, classified as either a miRNA, siRNA or piRNA based on biogenesis, and found to regulate gene expression through association with an Argonaute family member (reviewed in [1]). However, recently it has been shown that bacteria can use CRISPR RNAs as a form of adaptive immunity [2] and that a bacterial Argonaute associates with small RNAs preferentially derived from plasmids [3], suggesting that RNA interference may be more ubiquitous than previously appreciated. Moreover, careful analysis of deep sequencing libraries has revealed many reads that cannot be assigned to known small RNAs, and instead map to mRNAs, snoRNAs, rRNAs, tRNAs or others (reviewed in [4]), suggesting the existence of previously unappreciated classes of small RNAs, some of which appear to be conserved among all domains of life.

The best studied of these new small RNAs are fragments of tRNAs that correspond to half of a mature tRNA. First described in *Escherichia coli* as a response to bacteriophage infection [5], these fragments have been observed in numerous organisms and are commonly referred to as tiRNAs (reviewed in [6]). These molecules are known to accumulate during stress, are generated by Rny1 in yeast, angiogenin (ANG) in humans, and the 5’ halves have been shown to be capable of inhibiting protein translation in multiple organisms [7,8], while either the 5’ halves or 3’ halves could theoretically associate with RNase Z or RNase P, respectively, to slice target RNAs [9,10].

Distinct from tRNA halves are the less well studied small RNAs known as tRNA derived RNA fragments (tRFs). There are three types of tRFs recognized, those derived from the extreme 5’ and 3’ ends of mature tRNAs (tRF-5s and tRF-3s), and those that map to the 3’ trailer fragment of precursor tRNA transcripts (tRF-1s). These classes were first observed in LNCaP and C4-2 cells, and one tRF-1 was found to promote cell proliferation [11]. Soon after, numerous tRF-5s were observed in HeLa cell nucleoli deep sequencing, and these small RNAs were found to be weakly associated with Argonautes 1 and 2, and one was shown to be generated by DICER1 [12]. Consistent with these previous reports, tRF-3 and tRF-1 sequences were reported in HEK293 cells (referred to as Type I and Type II tsRNAs, respectively), and were shown to be primarily cytoplasmic [13]. This study also showed that tRF-1s were formed by RNase Z as expected, tRF-3s and tRF-1s preferentially associated with Argonautes 3 and 4 over 1 and 2, tRF levels could affect the efficacy of miRNAs and siRNAs, and a tRF-3 but not a tRF-1 could act in trans RNA silencing.

Since the initial classification of tRFs there have been multiple studies on tRFs in organisms ranging from archaea to humans, and these studies have been summarized in several recent reviews [14-16]. These reviews highlight the conflicting reports of the biogenesis of tRF-5s and tRF-3s, with some original reports on these molecules implicating DICER1, but a recent paper showing DICER1 is dispensable for the generation of most tRF-5s and tRF-3s [17]. Several recent papers have also shown that tRF-5s or tRF-3s can associate with Argonautes or participate in RNA silencing [18-20], while another paper argues tRF-5s cannot silence a reporter gene but rather function similarly to 5’ tRNA halves and inhibit translation [21]. Despite this growing literature on tRFs there are still concerns that tRFs could simply represent degradation products of their extremely abundant parental molecules, or concerns that the reads seen in deep sequencing are biologically relevant since it is known tRNA modifications can affect reverse transcriptase [22].

We have taken advantage of the recent explosion of small RNA-Seq data and novel methods of mapping the miRNA interactome to perform a meta-analysis of tRFs and provide insight into their properties. Our analysis of publicly available data sets clearly shows that tRFs are DROSHA-, DICER1-independent precisely generated fragments present in organisms ranging from bacteria to humans. We find that tRF-5s and tRF-3s, but not tRF-1s, are very abundant in AGO1, 3 and 4 photoactivatable-ribonucleoside-enhanced crosslinking and immunoprecipitation (PAR-CLIP) data and use canonical miRNA seed rules to associate with mRNAs. Analysis of AGO1 crosslinking, ligation, and sequencing of hybrids (CLASH) data suggests tRF-5s and tRF-3s may interact with thousands of different RNAs in human cells.

Despite the fact that tRFs are more evolutionarily conserved than miRNAs, are present in similar abundance, and are the only small RNA to display clear Argonaute sorting in humans, there is not a universally accepted nomenclature for tRFs or unique identifiers for different tRF sequences. As a result, it is possible for multiple labs to be working on the same tRF without noticing it, for example, cand45 in [12] is the same molecule as tRF-1001 in [11], or for a tRF to be misannotated as a miRNA [23]. To help the community study this new class of small RNA, we have created tRFdb [24], a relational database of tRNA derived RNA fragments, with all the tRF sequences which we have observed and unique identifiers. The names of datasets analyzed for each figure in this article are given in Table 1.

**Table 1:** **Name of datasets analyzed for each figure**

**Figure Name**	**Analyzed library**	
Figure 1A	GSM416733	
Figure 1B	GSM416733	
Figure 2A	GSM416733	HEK293
	GSM416753	HeLa
	GSM416754	U2OS
	GSM416755	143B
	GSM416756	A549
	GSM416757	H520
	GSM416758	SW480
	GSM416759	DLD2
	GSM416760	MCF7
	GSM416761	MB-MDA231
Figure 2B	GSM314552	Mouse-Esc
	GSM416732	Mouse-MEF
	GSM466487	Drosophila
	GSM604032	C.elegans
	GSM775340	S.cerevisiae
	GSM757894	S.pombe
	GSM1208316	R.sphaeroides
Figure 2C	GSM510432toGSM510435	Ovary
	GSM51043toGSM510439	Testes
	GSM510440toGSM510444	Brain
	GSM510445toGSM510456	Newborn
	GSM510457toGSM510460	E12.5
	GSM510465toGSM510468	E7.5
	GSM314552	ESC
Figure 3A-B	GSM416733	HEK293
Figure 4A-B	GSM314552	ESC_WT
	GSM314553	ESC_dcr--
	GSM314557	ESC_dgcr8--
Figure 4C-D	SRR029028	WT
	SRR029029	dcr-1
	SRR029030	dcr-2
Figure 4E	GSM466487	WT
	GSM466492	dcr-2
	GSM466496	r2d2
Figure 4F	GSM757894	WT
	GSM757897	dcr
Figure 4G	SRR207111	Whole-cell
	SRR207116	Nucleus
Figure 5A	GSM545212	AGO1
	GSM545213	AGO2
	GSM545214	AGO3
	GSB545215	AGO4
Figure 5B	GSM545212, GSM545213, GSM545214 and GSB545215 combined	
Figure 5C	GSM545212 dataset and the 17,319 CCRs reported in Hafner et al. [36].	
Figure 6	GSM1219487, GSM1219488, GSM1219489, GSM1219490, GSM1219491, GSM1219492 combined	
Figure 7	GSM1219487, GSM1219488, GSM1219489, GSM1219490, GSM1219491, GSM1219492 combined	

## Results

### tRFs are created by specific cleavage sites

**Figure 1:**
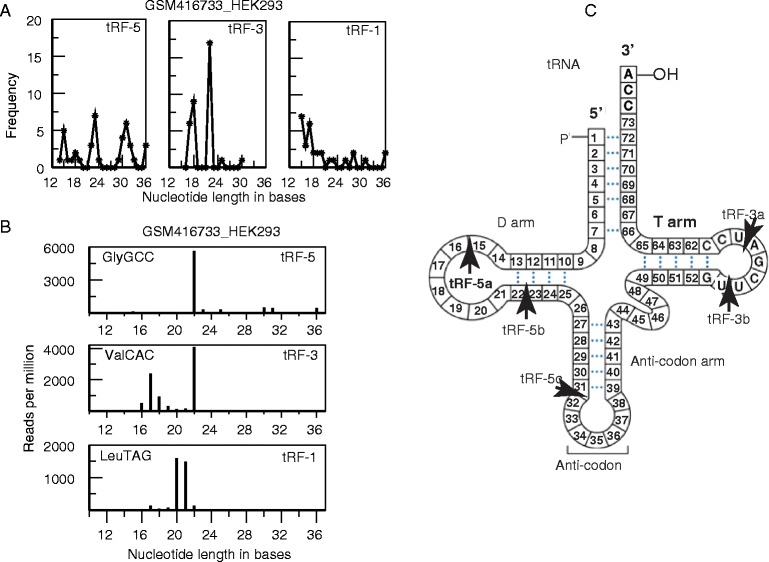
**Non-random mapping of small RNAs (tRFs) on tRNA genes (HEK293 human cell line).** **(A)** Numbers of unique tRFs that were present at a minimum of 20 reads per million are plotted against length of the tRF. **(B)** Length distribution of reads that mapped to a specific tRF-5 (GlyGCC), tRF-3 (ValCAC) and tRF-1 of (LeuTAG). **(C)** Illustration of a mature tRNA showing the cut sites that would generate the different subclasses of tRF-5s and tRF-3s.

We mapped small RNA reads from HEK293 cells to a collapsed tRNA gene [see Additional file 1: Figure S1] and, as expected, observed large numbers of reads that mapped to either the 5’ end, 3’ end or trailer sequence, corresponding to tRF-5s, tRF-3s and tRF-1s. Surprisingly, when we plotted the frequency of unique tRF reads of different lengths (Figure 1A) we observed three peaks for tRF-5s at ~15, ~22 and ~32 nts, and two peaks for tRF-3s at ~18 and ~22 nts. To the best of our knowledge, these distinct populations of tRF-5s and tRF-3s have never been reported before. The 5’ ends of ‘3’ tRNA halves’ have 5’ hydroxyl rather than a 5’ phosphate and are biochemically different from tRFs and other small RNA. In addition, the tRNA halves are cleaved in the middle of the anticodon loop producing a 34 to 36 nt fragment making it possible to distinguish them from most tRF-5s (<32 bases) with 3’ ends clearly in the stem and not in the anticodon loop itself. As shown in Figure 1C, we have developed a nomenclature for these subclasses of tRF-5s and tRF-3s: 3’ cleavage at +5 (tRF-5a), +22 to +24 (tRF-5b) and +30 to +32 (tRF-5c), 5’ cleavage at +55 (tRF-3b) and +59 to +60 (tRF-3a). The tRF-5 cleavage sites are in the D loop, D stem or the 5’ half of the anticodon stem, while the tRF-3 cleavage sites are both in the TΨC loop. These tRF subclasses are seen in all human data sets analyzed from [25] and are also conserved in mice, but become less distinct further down the evolutionary tree [see Additional file 1: Figure S2].

Most of the tRF-1s observed in this data set are 15 to 22 bases long and always begin at the end of the tRNA gene sequence which becomes a mature tRNA, and end with a RNA polymerase III (RNA pol III) transcription termination signal (UUUUU, UUCUU, GUCUU or AUCUU) [26,27]. Because the termination signal occurs at different locations in each pre-tRNA, tRF-1s are predicted to vary in length and, as expected, we found a broad length distribution (Figure 1A).

It is important to note that when looking at reads that map to a single tRNA, the peaks become much sharper (Figure 1B). The precision with which individual tRFs are generated strongly suggests that tRFs are not generated by random exonucleolytic digestion of longer precursors. In addition, because the method of small RNA sequencing in these data sets requires reverse transcriptase to read through the tRF into the adaptor sequence, tRNA modifications would, if anything, lower the number of reads that we are observing, not create artificial short sequences.

### tRFs in different cell lines, organisms, and tissues

**Figure 2:**
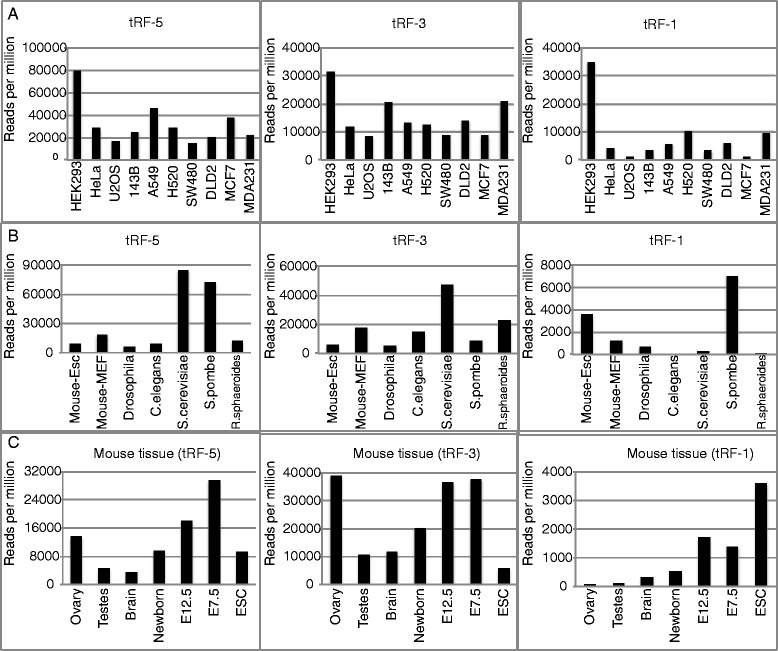
**Presence of tRFs in bacteria to human. (A)** Frequency of the three types of tRF in different human cell lines. tRF alignments that start with the first or second base of tRNA were collated as tRF-5 and whose 3’ end mapped to the 3’ end of tRNA and have a CCA at their 3’ end were categorized as tRF-3. tRFs whose 5’ end matched with the first or second bases of the 3’ trailer sequence of a tRNA were categorized as tRF-1. The number of tRF-5, tRF-3 and tRF-1 mapped in each cell line was normalized with the total number of reads in the analyzed library. **(B)** Shows the frequency of tRF-5, tRF-3 and tRF-1 in mouse embryonic stem cells, mouse cell line NIH3T3, *D. melanogaster*, *C. elegans*, *S. cerevisiae*, *S. pombe* and *R. sphaeroides.* **(C)** tRF expression in different mouse tissues and embryonic stem cells (ESC).

We next wanted to compare read counts for tRFs in cell lines other than HEK293 cells. We can observe read counts for all three classes of tRFs for all cell lines in the [25] data sets (Figure 2A). In general tRF-5s were present in higher abundance than tRF-3s, and tRF-3s were more abundant than tRF-1s.

To see if tRFs are present in other species we analyzed the publicly available small RNA data of mice [28], *Drosophila melanogaster* [29], Caenorhabditis *elegans* [30], Schizosaccharomyces *pombe* [31], *Saccharomyces cerevisiae* [32] and the bacterium *Rhodobacter sphaeroides* [3]. tRF-5s and tRF-3s are observed in all the species (Figure 2B). However fewer tRF-1s were observed in *Drosophila* (approximately 500 Reads Per Million (RPM)) and very few in *C. elegans*, *S. cerevisiae* and *R. sphaeroides*, although about 7,000 RPM of tRF-1s were detected in *S. pombe*.

The lower abundance of tRF-1s in the lower eukaryotes and absence in bacteria could be explained if the 3’ trailer sequences of pre-tRNAs were not in the 14 to 36-nucleotide range that was selected for cloning and sequencing. Indeed, 14 to 36 base-long pre-tRNA trailer sequences are ten-fold fewer in *C. elegans* and *S. cerevisiae* compared to human and mouse [see Additional file 1: Figure S3], which could account for the fewer tRF-1s in the small RNA libraries from these species. However, *Drosophila* has comparable numbers of 3’ trailers in the correct size range, and yet yielded fewer tRF-1, while *S. pombe* had fewer 3’ trailers in the correct size range and yielded a large number of tRF-1 clones. Thus, some factor other than the possible number of 3’ trailers in the correct size range, such as protein binding partners, helps determine how many tRF-1s are stable and identifiable in each species.

All the analyses of mammalian tRFs until now have been performed against RNA extracted from cell lines. To investigate if tRFs are also expressed in normal mammalian tissues, we analyzed the small RNA isolated from adult mouse ovary, testis and brain, and from mouse embryos and embryonic stem cells [28,33]. tRFs are present in all the tissues analyzed (Figure 2C), but the tRF-5s and tRF-3s were two- to five-fold less abundant in testes and brains compared to embryos, but as abundant in ovaries as in embryos. In contrast, the tRF-1s were less abundant in adult tissues with the highest level seen in brain, and that, too, was five- to twelve-fold less that in mouse embryos and embryonic stem cells.

### All tRNAs do not produce three tRFs, and not all tRFs are equally abundant

To determine if all tRNA genes produce all three types of tRFs and, if they do, whether the tRFs are in comparable abundance, we selected those tRNA genes where a tRF-1 was detected in HEK293 cells at >20 RPM. A given tRF-1 has a unique sequence that can be assigned to a specific tRNA gene. When the 5’ and 3’ ends of more than one tRNA gene are identical in sequence, we classify them as a tRNA family. Thus, we compare the cloning frequency of a specific tRF-1 with that of the tRF-5 or −3 derived from the corresponding family of tRNA genes. A tRNA family represents a group of genes encoding the same tRF-5 or −3 sequences. These could include tRNA isoacceptors but are not necessarily so.

**Figure 3:**
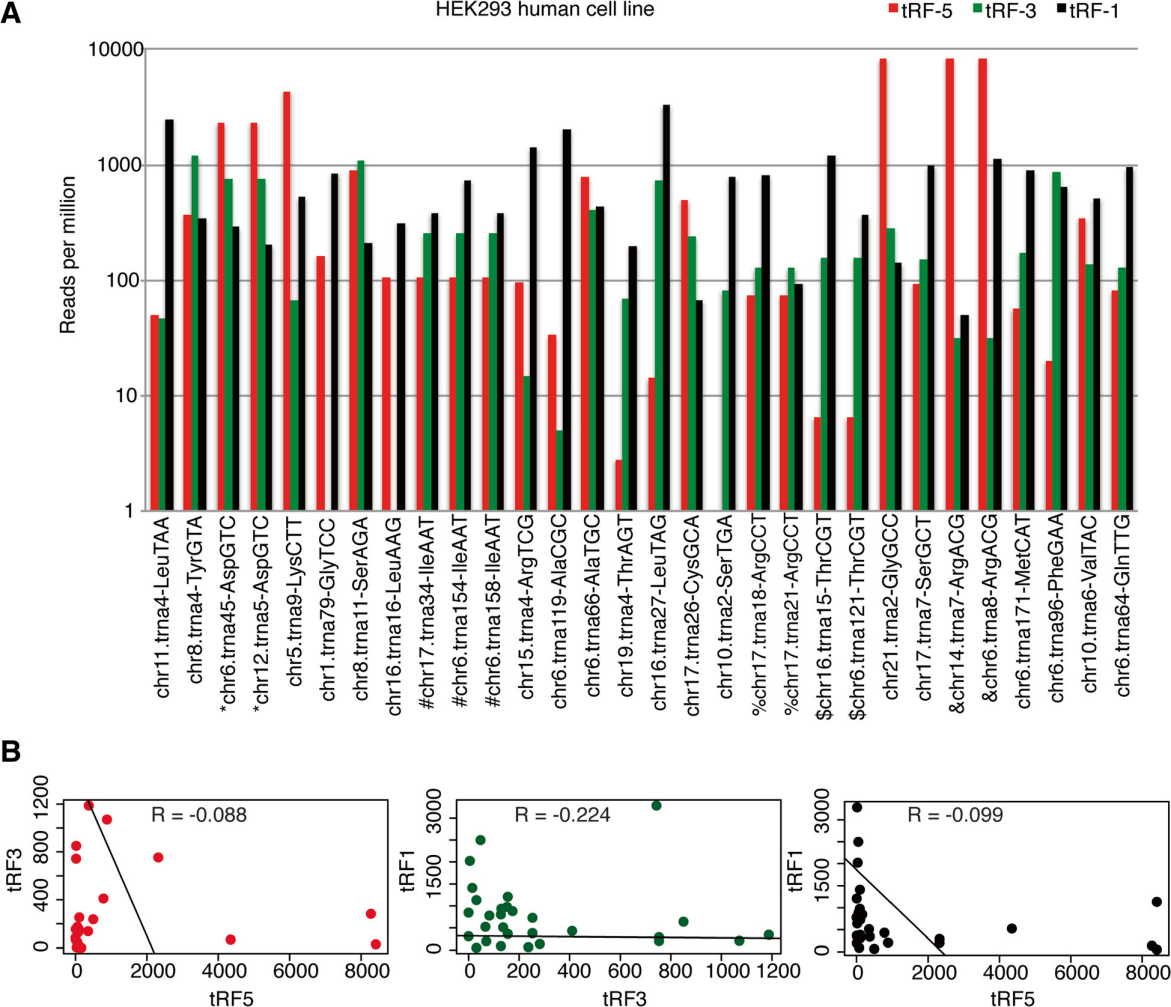
**A given tRNA does not yield tRF-5, −3 and −1 at equal abundance. (A)** Number of reads per million of tRF-5s, tRF-3s and tRF-1s from selected tRNA genes. The tRNA genes were selected on the basis of tRF-1s that had >20 reads per million in HEK293 human cell line library. The tRF-1s were compared with tRF-5s and -3s from the same tRNA, regardless of whether the tRF-5 or −3 was derived from that specific tRNA gene or other members of the tRNA gene family. The duplicated tRNA genes (tRNAs with the same anticodon) are marked with special character “*”, “#”, “$”, “%” and &. **(B)** Scatter plots of tRF-3s versus tRF-5s, tRF-1s versus tRF-3s, and tRF-1s versus tRF-5s for the tRNA genes shown in **A** along with Pearson correlation coefficients.

The sequencing frequencies of these matched sets of tRFs were plotted (Figure 3A). Not all the tRF types are detected for a given tRNA gene and family. For example, tRF-5-Ser^TGA^ or tRF-3-Gly^TCC^ or -Leu^AAG^ are selectively absent even though tRF-1 were detected in all three cases.

When all three tRFs from a given tRNA gene or family are detected, their cloning frequencies are not similar. For example, tRNA4-leu^TAA^ produces a tRF-1 that is nearly 40- to 50-fold more abundant than the tRF-5 or −3 generated from the Leu^TAA^ tRNA family, and the Pearson correlation coefficients between tRF-5s and tRF-3s (R = −0.088), tRF-3s and tRF-1s (R = −0.224) and tRF-5s and tRF-1s (R = −0.099) are very low (Figure 3B). The lack of a correlation between the concentrations of tRF-5, −3 or −1 from a given tRNA gene (or family) further supports the hypothesis that tRFs are non-random, stable products derived from specific tRNAs and pre-tRNAs.

### Processing of tRFs is distinct from miRNA biogenesis

**Figure 4:**
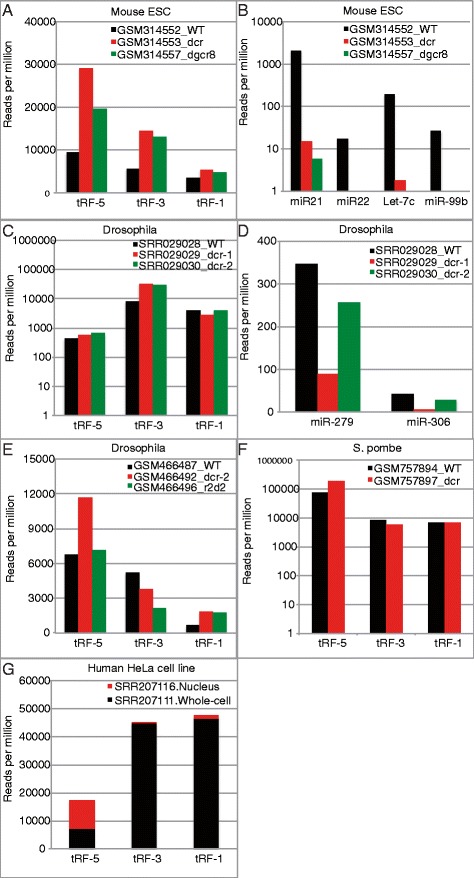
**Processing of tRFs is distinct from miRNAs and tRF-3 and tRF-1 are mostly cytoplasmic. (A)** tRF read counts in wild type, *dicer1* −/−, and *dgcr8*−/−mouse ES cells. **(B)** Same data sets as A, but read counts of various miRNAs are shown. **(C)** tRF read counts in *Drosophila* S2 cells either mock, *dicer-1* dsRNA, or *dicer-2* dsRNA treated. **(D)** Same data sets as C, but read counts of two miRNAs are shown. **(E)** tRF read counts from fly heads of either wild type, *dicer-2* mutant, or *r2d2* mutant flies. **(F)** tRF reads in wild type or *dcr1* delta *S. pombe*. **(G)** tRF read counts in HeLa cell nuclear fractionation or whole cell.

To study the role of DICER1 in the generation of tRFs, we investigated the high throughput sequencing data of short RNAs from the wild type and *dicer1* mutants isolated under similar conditions from the same experiments. Such data were available for three species, that is, mouse [28], *S. pombe* [31] and two data sets for *Drosophila* [34,35]. Mutation of DICER1 (or Dicer-1 in *Drosophila*) did not significantly decrease the expression of any of the three classes of tRFs in mice (Figure 4A), *S. pombe* (Figure 4F) and *Drosophila* (Figure 4C and E), in contrast to the nearly hundred-fold suppression of the cloning frequency of several microRNAs in mouse (Figure 4B) and three- to twenty-fold suppression in *Drosophila* (Figure 4D). DGCR8 (an essential partner for the Microprocessor complex that cleaves pri-miRNA to generate pre-miRNA) was similarly dispensable for tRF generation (Figure 4A). Dicer-2 and the double strand RNA binding protein R2d2 are involved in the biogenesis of siRNA in *Drosophila*. Mutation of *dicer-2* or *r2d2* did not decrease the expression of tRF-5 or −1 either (Figure 4D-E). Although *r2d2* mutation decreased tRF-3 levels to about 40%, in the context of all the other mutants, we conclude that the proteins involved in generating canonical miRNAs or siRNAs are dispensable for the generation of tRFs in mice, *Drosophila* and *S. pombe*.

### tRF-5s are nuclear while tRF-3s and -1s are cytoplasmic

To determine the cytoplasmic or nuclear location of tRFs we analyzed the small RNA of 18 to 30 bases isolated separately from nuclei and whole cell fraction of HeLa cell line [36] (Figure 4G). The tRF-5s were equally abundant in the whole cell and nuclear fractions, suggesting that they are mostly present in the nucleus, consistent with the observation of large numbers of tRF-5s in Hela cell nucleoli [12]. tRF-3s and tRF-1s were much more abundant in the whole cell fraction compared to the nuclear fraction suggesting that both species are almost exclusively in the cytoplasm, which is consistent with the findings of [13]. The specific subcellular localization of the classes of tRFs raises questions about their biogenesis and functions. We note that in most analyzed short RNA libraries we see an abundance of tRFs in the order tRF-1 < tRF-3 < tRF-5 (Figure 2A); however, the reverse trend was observed in short RNA libraries generated from the whole cell and nuclear fractions (Figure 4G). This may be due to variations in the protocol used by the lab that produced these libraries, but the effect should be the same on both the nuclear and whole cell libraries. Thus, we do not expect such variations to uniquely enrich tRF-5 in the nuclear fraction compared to tRF-3 or tRF-1.

### tRF-5s and tRF-3s associate with AGO1, 3, and 4

**Figure 5:**
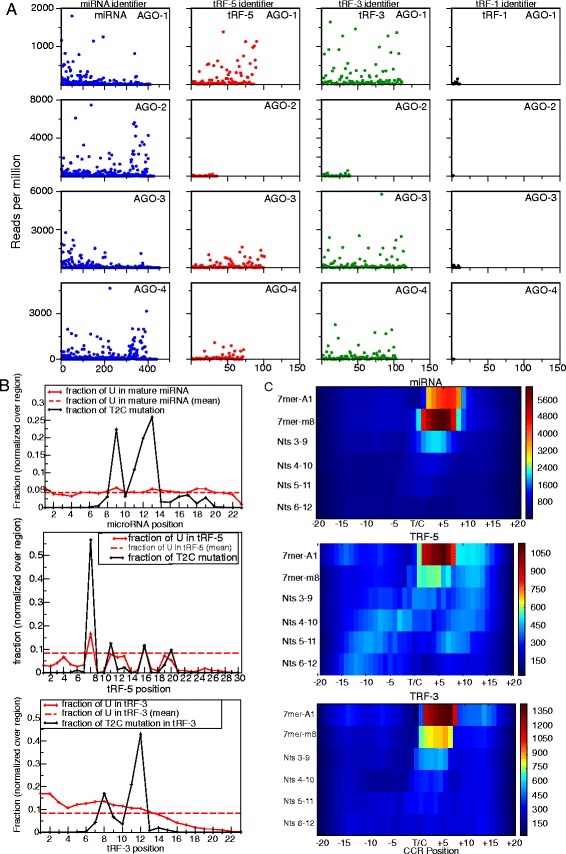
**PAR-CLIP analysis of miRNAs and tRFs. (A)** Read counts for miRNAs, tRF-5s, tRF-3s and tRF-1s in AGO 1 to 4 PAR-CLIP data from Hafner *et al* [37]. Each microRNA and tRF is given an identifying number. The expression level of each microRNA and tRF is shown on the Y-axis and the assigned number is shown on the X-axis. **(B)** Normalized positional T to C mutation frequencies for miRNA, tRF-5 and tRF-3 reads found in the AGO 1 to 4 PAR-CLIP data. **(C)** Matches of canonical and noncanonical seeds of the 50 most abundant miRNAs, tRF-5s or tRF-3s seen in the AGO1 dataset to the 17,319 CCRs reported in Hafner *et al*.

We investigated the association of tRFs with human Argonautes by analyzing the human AGO1, 2, 3 and 4 PAR-CLIP data isolated from HEK293 cell lines [37]. In PAR-CLIP, when the 4-thiouridine is crosslinked to the protein of interest, it often becomes mutated to a cytidine during library preparation. Positional T to C mutation analysis of the data provides information about the RNA-protein interaction. In the presented analysis we allow 1 T/C mutation and give preference for perfect mappings (see Methods for details). Read counts for tRF-5s and tRF-3s are comparable to miRNAs for AGO1, AGO3 and AGO4, but are nearly absent in AGO2, while there are almost no read counts for tRF-1s for all four Argonautes (Figure 5A). Since this is the first time a class of small RNA has been reported to show differential human Argonaute sorting, we were interested if we could observe this trend in other data sets. AGO1 and AGO2 HEK293 cell PAR-CLIP was also performed by [38] and analysis of this data again showed this same pattern (data not shown). Unfortunately, we are unaware of any mouse AGO1, 3 or 4 CLIP or PAR-CLIP data, so we could not repeat this analysis for mice, but we do note that only very small numbers of tRFs are seen in mouse AGO2 CLIP data [39] (data not shown).

### tRF-3s and tRF-5s bind to human Argonautes like miRNAs

As reported in [37], miRNAs are crosslinked to the AGO protein at specific positions, namely positions 9 to 13, and this is borne out by a high T to C mutation frequency at these positions and very low mutational frequency at other positions, particularly the first 7 positions, that constitute the ‘seed’ and are involved in base-pairing with the target RNA. We first checked if we could replicate these results with our algorithms (Figure 5B), and we were able to detect a high percentage of T to C mutations at positions 9 to 13 for miRNAs and a very low frequency at the first 7 positions. We next checked whether tRF-3s display a similar pattern of mutations since this would indicate a similar binding mode and perhaps function. As with miRNAs, we saw a very low mutation frequency for the first 6 positions and peaks between positions 8 to 12. The slight difference in mutational frequencies between miRNAs and tRF-3s could be due to a sampling bias since tRF-3s contain fewer Ts than miRNAs, and the Ts that are present are not as randomly distributed as in miRNAs. Alternatively, this difference could indicate a biologically relevant distinction in the way tRF-3s interact with Argonautes. The T to C mutational frequency of tRF-5s also shows protection from cross-linking of the first six residues (Figure 5B (lower panel), suggesting that these bases are facing away from the Argonaute in an orientation suitable for binding to a target RNA, that is, they represent a seed region. The maximal T-C change is observed at base 7, and not at bases 9 to 13, unlike what is observed with microRNAs and tRF-3s. This is partly because tRF-5s are enriched in Us at base 7, but may also be because some of them (tRF-5c, Figure 1A) form a slightly different complex with Argonautes because they are longer at 32 bases than miRNAs and tRF-3s.

### tRF-3s associate with target RNAs via canonical seed sites

In addition to miRNAs being cross-linked at specific positions in PAR-CLIP, target RNAs are also preferentially cross-linked at a certain position with respect to the RISC complex: in the middle of the complex immediately preceding the sequence annealed to the microRNA seed. This information was used in [37] to generate 17,319 crosslink-centered regions (CCRs) of RNAs present in the PAR-CLIP data. CCRs are 41 nt long sequences centered at the T that showed the highest T to C frequency. They demonstrated that the reverse complement of known miRNA seeds is enriched in CCRs directly following this central cross-linked T. We reproduced this observation by taking the 50 most abundant miRNAs in AGO1 PAR-CLIP data and scanning the 17,319 CCRs with different seed definitions (Figure 5C). As expected, the canonical seed sites 7mer-A1 (target has an A at nucleotide position 1 and matches positions 2 to 7 of microRNA) and 7mer-m8 (target matches positions 2 to 8 of microRNA) produce the largest number of matches, and at the expected position in the CCRs, immediately downstream from the cross-linked T. The less canonical seed sequences, such as bases 3 to 9 of the microRNAs, produce fewer matches with the CCRs, and microRNA positions that have never been recognized as seeds produce even fewer.

Given that we saw similar patterns of T to C mutations for tRF-3s and miRNAs, we were emboldened to test whether the 5’ ends of tRF-3s act as seeds to select matching target CCRs, with the match at a location in the CCR that is immediately downstream of the central cross-linked T. As with miRNAs, the two best seed definitions of tRF-3s that matched CCRs are the canonical 7mer-A1 and 7mer-m8 (Figure 5C). However, while miRNAs seemed to show a preference for the 7mer-m8 site over the 7mer-A1 site, tRF-3s had the opposite preference. Again, this could represent sampling bias, or it may hint at a RNA-induced silencing complex (RISC) that is fundamentally different. Significantly, the best complementary matches to the tRF-3 seeds were also immediately downstream of the central T in the CCRs, just as seen with the miRNAs. Seeds of tRF-5s also show matches above background with canonical sites in the CCRs (Figure 5C), but tRF-1s do not show matches above background [see Additional file 1: Figure S4].

### CLASH data indicates tRF-3s and tRF-5s target thousands of RNAs

**Figure 6:**
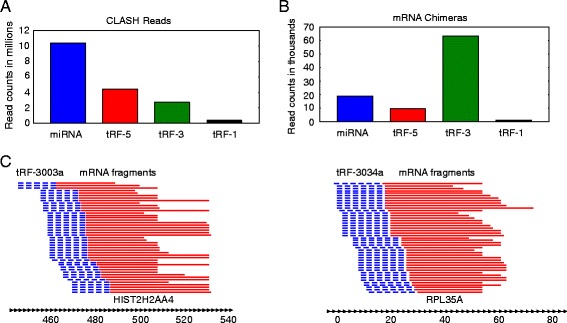
**tRF-mRNA chimeras are abundant in AGO1 CLASH data. (A)** Numbers of CLASH reads that started with a perfect match to a miRNA, tRF-3, tRF-5 or tRF-1 and deemed to not be pre-miRNA, tRNA or a RNA in Ref-Seq. **(B)** Numbers of miRNA, tRF-5, tRF-3 or tRF-1 chimeras with mRNAs. **(C)** Alignments of the mRNA portion of the 45 most abundant reads for the tRF-3003a-HIST2H2AA4 interaction and the tRF-3034a-RPL35A interaction to the corresponding mRNA. The tRF-3 portion of the reads is depicted in dashed blue while the mRNA fragment is depicted in red. CLASH, cross-linking, ligation, and sequencing of hybrids.

**Figure 7:**
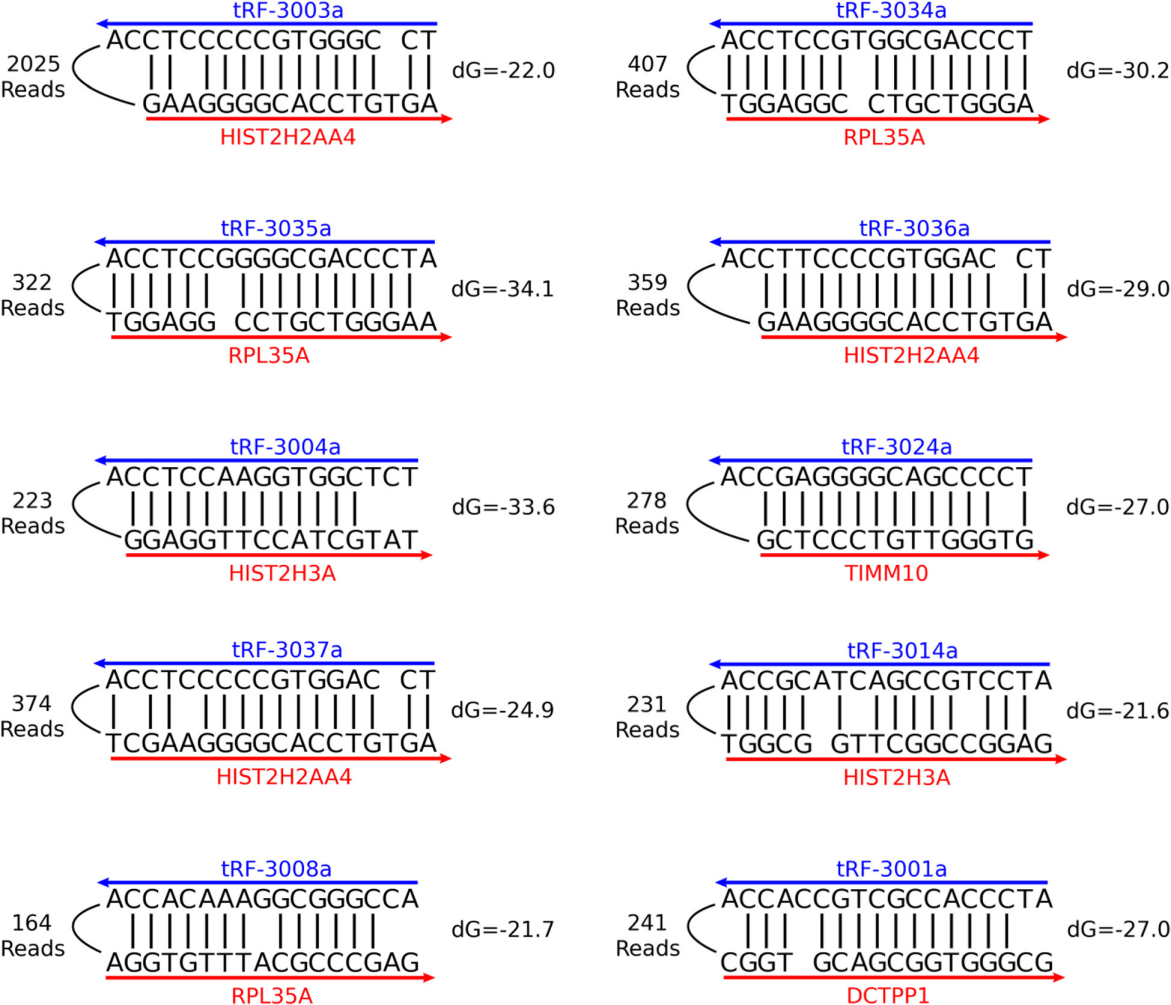
**Examples of tRF-3-mRNA CLASH chimeras.** The most abundant read of 10 of the most prevalent tRF-3-mRNA interactions found in our analysis were analyzed with mfold’s RNA Folding Form using default settings. The output of mfold is represented with the tRF depicted 3’ to 5’ and the mRNA sequence 5’ to 3’. For each interaction the number of reads supporting the interaction is shown to the left, and the delta G from mfold is shown to the right. CLASH, cross-linking, ligation, and sequencing of hybrids.

CLASH is a new technique that has recently been used to study the AGO1-miRNA-target RNA interactome in HEK293 cells [40]. Briefly, the technique is similar to CLIP, but with the addition of a ligation step that connects the 3’ end of the AGO bound small RNA to the 5’ end of the target RNA. To analyze this data we found reads that started with either a miRNA or tRF, and then performed blastn with the rest of the sequence against human Ref-Seq RNA (see Methods). In our analysis we see 187 HOXC8-mir-196a/b chimeric reads, which corresponds closely to the 191 chimeric reads identified by Helwak *et al* [40]. Surprisingly, despite miRNAs being more abundant, we saw more tRF-3-mRNA chimeras than miRNA-mRNA chimeras (Figure 6A-B). We also observed numerous tRF-5-mRNA chimeras, but very few tRF-1-mRNA chimeras, which is consistent with our PAR-CLIP analysis (Figure 6B). Manual observation of some of the more abundant tRF-3-mRNA chimeras shows nice clustering of the mRNA portions of the reads (Figure 6C). Examples of some of the most abundant tRF-mRNA interactions are shown with their predicted mfold structures (Figure 7), and a list of all tRF-mRNA chimeric reads can be found in Additional file 2.

## Discussion

The recent increase in small RNA-Seq data and novel methods to investigate the miRNA-interactome allowed us to perform a detailed analysis of the properties of tRFs. We have shown that tRFs are very precisely generated fragments that are present in all cell lines investigated and in organisms ranging from humans to bacteria. Using well-established PAR-CLIP data we showed that tRF-5s and tRF-3s clearly associate with human Argonautes 1, 3 and 4, but show little to no association with AGO2. Although Argonaute sorting is common in some organisms such as plants, this has not been seen in humans before. tRF-5s and tRF-3s also match clusters observed in PAR-CLIP data with canonical seed rules, indicating that AGO-tRF complexes are able to associate with RNAs. CLASH analysis confirmed this hypothesis by the observation of large numbers of tRF-mRNA chimeras.

This investigation raises many questions about the biology of tRFs and small RNAs in general. If DICER1 and DROSHA are not involved in tRF generation then which proteins are? Do all organisms generate tRFs by the same pathway? Do tRFs associate with Argonautes in other organisms? Is the lack of association with AGO2 telling us something about RISC assembly in humans? And will the traditional methods for studying miRNAs (such as antisense RNAs) be applicable to a small RNA whose parental RNA is one of the most abundant RNAs in the cell?

These questions are too much for any one group to answer, but it is tempting to speculate as to the possibilities. tRNAs are heavily modified and it is known that some of these modifications affect tRNA stability. Indeed, lack of a specific tRNA modification has been shown to increase tRNA half generation [41]. This suggests that there may be other modifications which can affect tRF-5 or tRF-3 generation.

The matches to PAR-CLIP clusters and the large number of CLASH chimeras point to a role for tRFs in RNA silencing. In fact, it is known that a large number of CLIP-Seq clusters are not able to be assigned to miRNA seeds [42]. Thus far, the scientific community has explained this fact by proposing noncanonical seeds, such as those that contain bulges, but it may be that these orphan clusters are targeted by tRFs. However, it is important to keep an open mind for the function of tRFs. For example, a recent publication showed that a tRF-3 which our lab previously identified is able to serve as a primer for HTLV-1 reverse transcriptase [43]. In addition, some of the most abundant tRF-3 chimeras we observe are with histone mRNAs. Histone mRNAs are known as the only mRNAs in the cell to not contain a poly-A tail, thus these interactions cannot result in traditional degradation of the mRNA target. However, the binding site for a large number of the interactions is very close to the stem loop, indicating the tRF-3s could compete with Stem-loop binding protein and affect the mRNA stability via this mechanism.

In addition, although miRNA-Argonaute complexes are traditionally thought to function in the cytoplasm, increasingly diverse functions for Argonautes in the nucleus continue to be discovered (reviewed in [44]). For example, there have been recent reports of transcriptional gene silencing by short RNAs and regulation of alternative splicing by Argonaute and related PIWI proteins in mammals. The presence of tRF-5s in the nucleus and the association of tRF-5s with Ago1, Ago3 and Ago4 suggest that tRF-5s may participate in these processes.

Although there has been a steady increase in tRF literature since their discovery, our current knowledge of tRFs clearly pales in comparison to other small RNAs. We have shown that tRFs display similar properties to miRNAs, and given the importance of miRNAs in processes ranging from development to cancer, it is not far-fetched to imagine equally important functions for tRFs.

## Conclusions

tRFs are a newly discovered class of small RNA that are highly abundant in different human cell lines, mouse tissues and organisms ranging from bacteria to humans. Individual tRFs show a narrow size distribution, suggesting that the fragments are precisely generated and not degradation products of tRNAs. Mutation of different components of the miRNA biogenesis pathway does not have an effect on tRF levels, and tRFs are seen in organisms that do not contain miRNAs, indicating tRF generation is distinct from miRNA biogenesis. In human HEK 293 cells tRF-5s and tRF-3s are associated with Argonautes 1, 3 and 4 as evidenced by PAR-CLIP data. These tRFs contain seed sequences, which match the central portion of large numbers of CCRs. This observation, along with the finding of thousands of tRF-mRNA chimeras in CLASH data, indicates tRF-3s and tRF-5s can target RNAs in a manner similar to miRNAs.

## Methods

### Analysis of the small RNA data

The data analyzed in this manuscript were downloaded from either the GEO database [45] or NCBI SRA database [46]. We considered only those sets of high throughput sequencing data where small RNAs of 14 to 36 bases long were size selected and then sequenced. For each dataset we looked for the processed sequence along with its cloning frequency. In case of non-availability of processed data, the raw data were used to generate the unique sequence and its cloning frequency. The adaptor sequences from the raw data were removed using the ‘Cutadapt’ (version 1.0) program [47].

### Building and mapping of small RNA on ‘tRNAdb’

Information about the tRNA genes for each species (Human hg19; Mouse mm9; Drosophila dm3; C. elegans ce6; S. cerevisiae sacCer1; S. pombe schiPomb1) was downloaded from the ‘Genomic tRNA database’ [48]. For each tRNA gene the DNA sequences ranging from 100 bases upstream of the start of mature tRNA to 200 bases downstream of the end of mature tRNA were extracted from the same genome assembly on which the tRNA gene coordinates were built. A species-specific tRNA database called tRNAdb’ was built. To find the tRNA-related RNA sequences in each library, the small RNAs were mapped on the species-specific tRNAdb, using BLASTn [49]. In general we considered only those alignments where the query sequence (small RNA) was mapped to the database sequence (tRNA) along 100% of its length. The blast output file was parsed to get information on the mapped position of small RNA on tRNA genes. We extracted all map positions where the small RNA aligned from its first base to the last base with the tRNA sequence allowing either one or no mismatch. Since ‘CCA’ is added at the 3’ end of tRNA by tRNA nucleotidyltransferase during maturation of tRNA [50], we allowed a special exception for the small RNA mapping to the 3’ ends of tRNAs in the tRNAdb allowing a terminal mismatch of < =3 bases. To remove any false positives, the small RNAs that mapped on to the ‘tRNAdb’ were again searched against the whole genome using blast search excluding the tRNA loci. Only those small RNAs were qualified as tRFs that mapped exclusively on tRNAdb.

### PAR-CLIP data analysis

We included the mature miRNA (miRNA:miRBase v20; genome-build-id: GRCh37.p5) and mRNA sequences in our previously built human specific tRNAdb that was used to query the expression level of tRFs and miRNA. We investigated tRF and miRNA expression with human Argonautes by analyzing the human Ago1 (GEO ID = GSM545212), 2 (GEO ID = GSM545213), 3 (GEO ID = GSM545214) and 4 (GEO ID = GSM545215) PAR-CLIP data isolated form HEK293 cell lines [37]. Data of all four small RNA libraries (AGO1 to 4) were combined together to examine the T to C mutation position and its frequency compared to wild type small RNA (miRNAs and tRFs). Sequence reads either mapped perfectly on miRNA or tRFs or mapped with one base mismatch were considered for T to C mutation analysis. Mismatched base and its position relative to the 5’ end of small RNA were collected for final analysis.

### CLASH data analysis

The miRNA, tRF-5, tRF-3 and tRF-1 analyses were performed separately. For the miRNA analysis, reads were found that started with a mature miRNA allowing no mismatches, giving preference to longer miRNAs. For the tRF-5 analysis, reads were found that started with a sequence that mapped to the first 14 to 33 nucleotides of a tRNA, allowing no mismatches, giving preference to longer tRF-5s. For the tRF-3 analysis, reads were found that started with a sequence that mapped to the last 17 to 23 nucleotides of a mature tRNA, allowing no mismatches, giving preference to longer tRF-3s. For the tRF-1 analysis, reads were found that started with a sequence that mapped to the first 14 to 33 nucleotides of a tRNA trailer, allowing no mismatches, giving preference to longer tRF-1s. For the miRNA analysis, the reads were confirmed to not be pre-miRNAs by running blastn, word size 7, default scoring matrix, against a database composed of miRNA hairpins from miRBase. For the tRF-5 analysis, the reads were confirmed to not be longer tRNA fragments or full length tRNAs by running blastn, word size 7, default scoring matrix, against a database composed of mature tRNA sequences. All reads were checked to not be RNA fragments by performing a blastn search against the human Ref-Seq database using blastn, word size 7, default scoring matrix, 20 maximum hits. Reads that had a hit which either overlapped 6 or more bases of the small RNA, or 6 or more bases of an 18 base minimal small RNA sequence for the longer small RNAs, and had an e-value less than or equal to .001, were discarded. For all analyses the portion of the read following the small RNA sequence was searched against the human RNA Ref-Seq database using blastn, word size 7, default scoring matrix, 20 maximum hits. Because blastn is a local aligner, a conservative approach was taken to adaptor removal. Adaptor was removed if a perfect match was found for 12 or more bases of the adaptor, or 1 mismatch for 21 or more bases of adaptor, or 2 mismatches for 26 or more bases of adaptor. Reads were considered chimeras if a hit was found within four nucleotides of the end of the small RNA and had an e-value less than or equal to .01. Because the search space can affect the e-value, reads still possibly containing adaptor sequence and having a borderline e-value underwent adaptor removal with Biopython’s pairwise2 local aligner and were blast searched again to get an updated e-value. Many reads matched more than one transcript in Ref-Seq. To identify the most likely transcript, every read of the CLASH data was searched against the human Ref-Seq database using blastn, word size 7, default scoring matrix, 20 maximum hits. All hits with an e-value less than .1 were tabulated. For the chimeric reads, the most likely transcript was deemed to be the transcript which was most abundant in the data. If a tie still occurred, NM transcripts were given preference to XM transcripts, XM given preference to NR, and NR given preference to XR. All chimeras whose most likely transcript was either NM or XM were deemed to be a small RNA-mRNA chimera. All such tRF chimeras are reported in Additional file 2, along with up to 19 other possible transcripts sorted by likelihood.

## Additional files

Additional file 1: Figure S1. Non-random mapping of small RNA (tRFs) on tRNA genes in HEK293 cell lines. tRNA gene co-ordinates were collapsed to 1–73 bases long mature tRNA. The scale 1 to 73 on the x-axis is the 1st to 73rd base of mature tRNA gene. The 5’ and 3’ ends of tRFs mapped on tRNA were recorded. The number of tRF ends that map to a specific base of tRNA locus is shown. The dotted lines predict the three types of tRFs. **Figure S2.** Non-random mapping of small RNA (tRFs) on tRNA genes in other species. The axes and other details are same as given in **Figure S1** legend. The number of tRF ends (5’ or 3’) mapped at each base given as reads per million in: mouse embryonic stem cells, mouse cell line NIH3T3, *D. melanogaster*, *C. elegans*, *S. cerevisiae* and *S. pombe*. **Figure S3.** Predicted length distribution of tRNA trailer sequences in different organisms. The computational prediction of length distribution of tRNA trailer sequences (potential tRF-3 s) in human, mouse, Drosophila, *C. elegans*, *S. cerevisiae* and *S. pombe*. **Figure S4.** Matches of canonical and noncanonical seeds of the 50 most abundant tRF-1s seen in the AGO1 dataset to the 17,319 CCRs reported in Hafner *et al.* [37]. Additional file 2:
**Excel file containing all tRF-mRNA chimeric reads observed in the CLASH data.** There are separate workbooks for tRF-1s, tRF-3s, and tRF-5s. The most likely to least likely mapping of the mRNA portion of the read is listed left to right with up to 20 transcripts listed.

## Abbreviations

CCR: crosslink-centered region
CLASH: cross-linking, ligation, and sequencing of hybrids
miRNA: microRNA
PAR-CLIP: photoactivatable-ribonucleoside-enhanced crosslinking and immunoprecipitation
RISC: RNA-induced silencing complex
RPM: reads per million
siRNA: small interfering RNA
tiRNA: tRNA-derived stress-induced fragments
tRF: tRNA-derived RNA fragment
