# Supplementary figures and images for: Meta-analysis of tRNA derived RNA fragments reveals that they are evolutionarily conserved and associate with AGO proteins to recognize specific RNA targets
Source: BMC Biol. 2014 Oct 1;12:78. doi: 10.1186/s12915-014-0078-0 (PMC4203973; doi:10.1186/s12915-014-0078-0)

Figure: S1

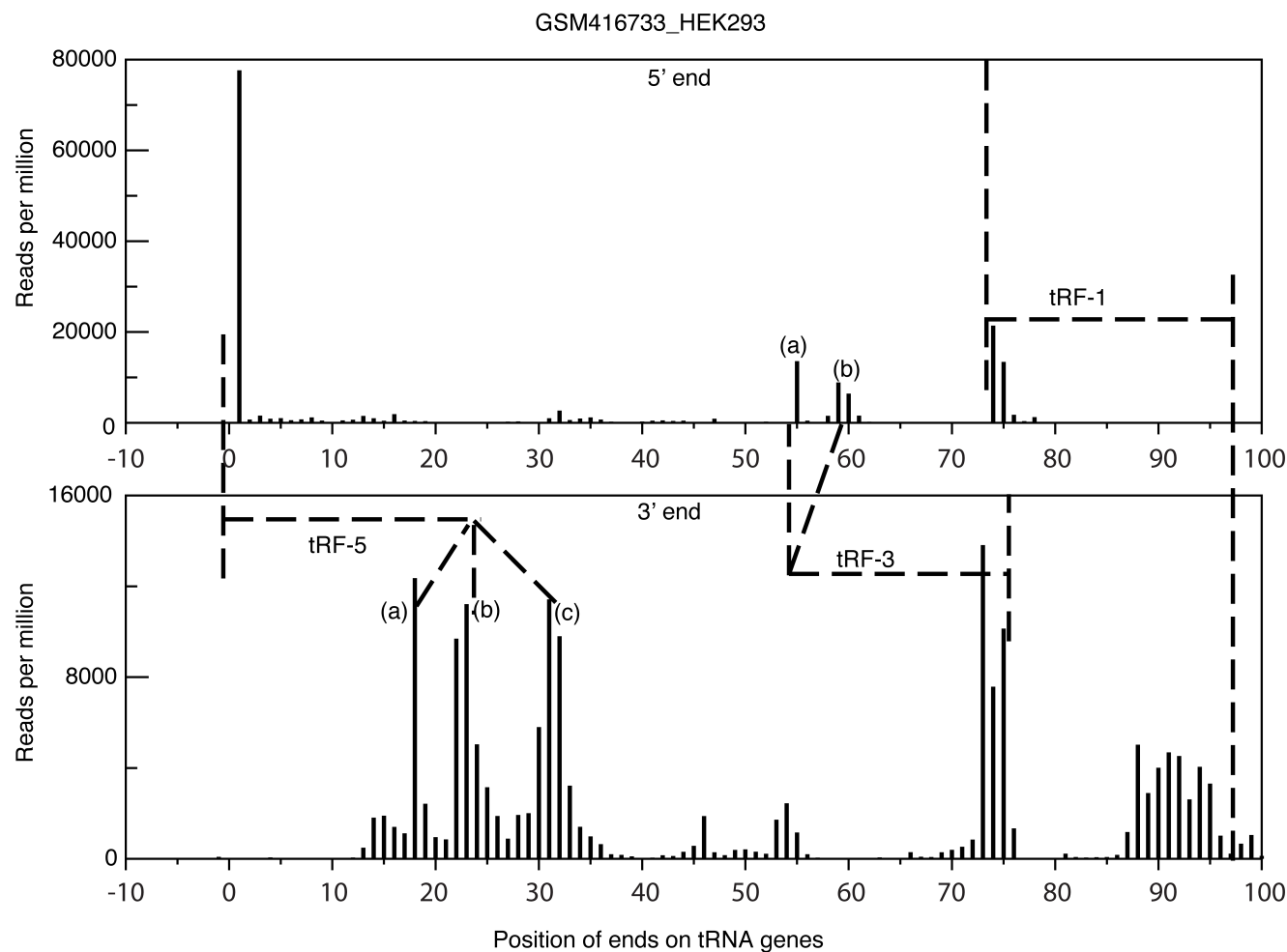

Figure: S2

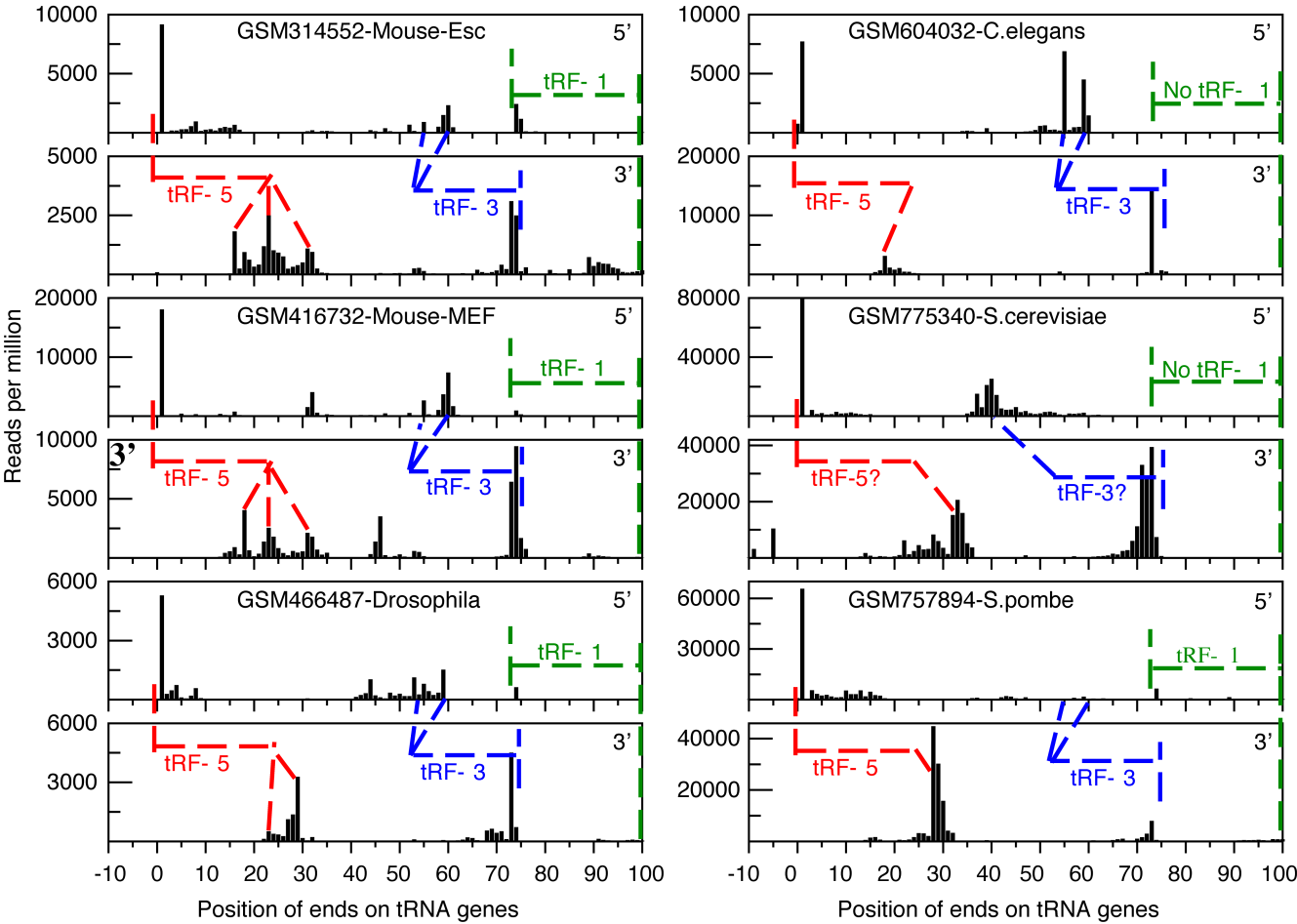

Figure: S3

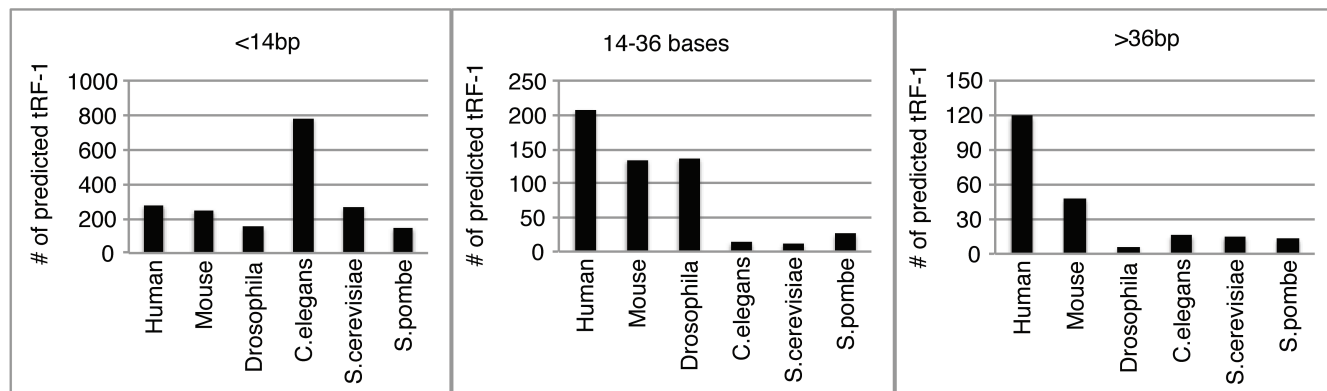

Supplement: Additional file 1: Figure S1. — Non-random mapping of small RNA (tRFs) on tRNA genes in HEK293 cell lines. tRNA gene co-ordinates were collapsed to 1–73 bases long mature tRNA. The scale 1 to 73 on the x-axis is the 1st to 73rd base of mature tRNA gene. The 5’ and 3’ ends of tRFs mapped on tRNA were recorded. The number of tRF ends that map to a specific base of tRNA locus is shown. The dotted lines predict the three types of tRFs. Figure S2. Non-random mapping of small RNA (tRFs) on tRNA genes in other species. The axes and other details are same as given in Figure S1 legend. The number of tRF ends (5’ or 3’) mapped at each base given as reads per million in: mouse embryonic stem cells, mouse cell line NIH3T3, D. melanogaster, C. elegans, S. cerevisiae and S. pombe. Figure S3. Predicted length distribution of tRNA trailer sequences in different organisms. The computational prediction of length distribution of tRNA trailer sequences (potential tRF-3 s) in human, mouse, Drosophila, C. elegans, S. cerevisiae and S. pombe. Figure S4. Matches of canonical and noncanonical seeds of the 50 most abundant tRF-1s seen in the AGO1 dataset to the 17,319 CCRs reported in Hafner et al. [37]. [file 12915_2014_78_MOESM1_ESM.zip › 12915_2014_78_figS1,figS2,figS3.pdf]

Figure S4:

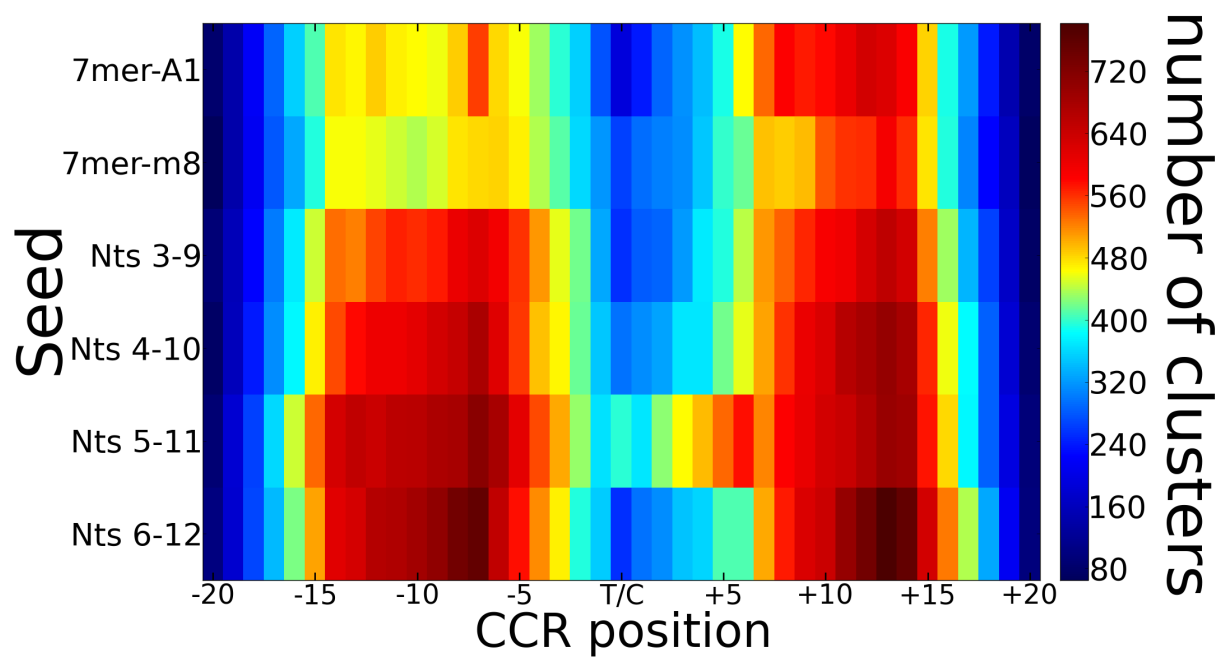

Supplement: Additional file 1: Figure S1. — Non-random mapping of small RNA (tRFs) on tRNA genes in HEK293 cell lines. tRNA gene co-ordinates were collapsed to 1–73 bases long mature tRNA. The scale 1 to 73 on the x-axis is the 1st to 73rd base of mature tRNA gene. The 5’ and 3’ ends of tRFs mapped on tRNA were recorded. The number of tRF ends that map to a specific base of tRNA locus is shown. The dotted lines predict the three types of tRFs. Figure S2. Non-random mapping of small RNA (tRFs) on tRNA genes in other species. The axes and other details are same as given in Figure S1 legend. The number of tRF ends (5’ or 3’) mapped at each base given as reads per million in: mouse embryonic stem cells, mouse cell line NIH3T3, D. melanogaster, C. elegans, S. cerevisiae and S. pombe. Figure S3. Predicted length distribution of tRNA trailer sequences in different organisms. The computational prediction of length distribution of tRNA trailer sequences (potential tRF-3 s) in human, mouse, Drosophila, C. elegans, S. cerevisiae and S. pombe. Figure S4. Matches of canonical and noncanonical seeds of the 50 most abundant tRF-1s seen in the AGO1 dataset to the 17,319 CCRs reported in Hafner et al. [37]. [file 12915_2014_78_MOESM1_ESM.zip › 12915_2014_78_FigS4.pdf]
